# First use of triply labelled water analysis for energy expenditure measurements in mice

**DOI:** 10.1038/s41598-022-10377-8

**Published:** 2022-04-15

**Authors:** Xing Wang, Dehuang Kong, Gertjan van Dijk, Harro A. J. Meijer

**Affiliations:** 1 Centre for Isotope Research (CIO), Energy and Sustainability Research Institute Groningen (ESRIG), University of Groningen, Nijenborgh 6, 9747 AG Groningen, The Netherlands; 2 Unit Behavioral Neuroscience, Groningen Institute for Evolutionary Life Sciences, University of Groningen, Nijenborgh 7, 9747 AG Groningen, The Netherlands

**Keywords:** Biological techniques, Endocrinology

## Abstract

The doubly labelled water (DLW) method is widely used to determine energy expenditure. In this work, we demonstrate the addition of the third stable isotope, ^17^O, to turn it into triply labelled water (TLW), using the three isotopes measurement of optical spectrometry. We performed TLW (^2^H, ^18^O and^17^O) measurements for the analysis of the CO_2_ production (r_CO2_) of mice on different diets for the first time. Triply highly enriched water was injected into mice, and the isotope enrichments of the distilled blood samples of one initial and two finals were measured by an off-axis integrated cavity output spectroscopy instrument. We evaluated the impact of different calculation protocols and the values of evaporative water loss fraction. We found that the dilution space and turnover rates of ^17^O and ^18^O were equal for the same mice group, and that values of r_CO2_ calculated based on ^18^O–^2^H, or on ^17^O–^2^H agreed very well. This increases the reliability and redundancy of the measurements and it lowers the uncertainty in the calculated r_CO2_ to 3% when taking the average of two DLW methods. However, the TLW method overestimated the r_CO2_ compared to the indirect calorimetry measurements that we also performed, much more for the mice on a high-fat diet than for low-fat. We hypothesize an extra loss or exchange mechanism with a high fractionation for ^2^H to explain this difference.

## Introduction

The doubly labelled water (DLW) method, first proposed by Lifson et al. in the middle of the last century, is a reliable, harmless and non-invasive method to determine the energy expenditure and body composition for humans and free-living animals^1–6^. In practice, a dose of known concentration water, highly enriched in the isotopes ^2^H and ^18^O, is introduced to the body, where the mixtures will quickly spread evenly through the body water pool and thus get diluted. The general principle of the method is based on the fact that hydrogen leaves the body through water turnover, while oxygen leaves both through water turnover and through respiratory CO_2_. The ^2^H and ^18^O abundances of body fluids are measured from the initial to the final time points, and then the isotope elimination rates can be determined. The difference between these two turnover rates is then proportional to the CO_2_ production (r_CO2_), which can be further converted to energy expenditure if the composition of the food intake is known^1,4,7–9^.

Although the basic theory is straightforward, several complications are involved when conducting the actual r_CO2_ calculation^2,4,10–14^. For example, we need to consider the oxygen isotope fractionation between CO_2_ and body water, as well as the fractionation between water and water vapour. These fractionation factors are well known from various laboratory experiments in the past^4,8,15–19^. However, certain aspects of the process are less well-known (and possibly variable), such as the level of (non-)equilibrium in the fractionation process during evaporative H_2_O loss, and the fraction of the water that leaves the body through evaporation. Different approaches for body water pool size calculations also matter for the final results^13,20^, although one can argue that the choice for the optimal way of calculation is clear from a principle point of view.

In the history of DLW, a series of calculation protocols have been used and studied. The comparison with other methods estimating energy expenditure methods, like the indirect calorimetry method, is highly valuable^13,21–23^.

In addition to the traditional DLW method, some researchers proposed or even used three isotopes instead of two (the combination of ^2^H, ^3^H and ^18^O or ^2^H, ^17^O and ^18^O), to trace isotope changes for quantifying the body water and CO_2_ fluxes^10,16,24^. The additional use of the third isotope reduces the analytical error, helps to check the data quality and in principle even gives the possibility to quantify the evaporative water loss fraction. However, ^3^H (tritium) is rare and radioactive, and therefore not attractive to complement ^2^H and ^18^O as a tracer. Addition of the other naturally occurring rare stable isotope of oxygen, ^17^O, was for a long time unattractive due to the complicated measurement methods when using Isotope Ratio Mass Spectrometry (IRMS), due to the mass overlap of the ^12^C^17^O^16^O and the ^13^C^16^O^16^O isotopologues. Avoiding this overlap could either be done by reduction of CO_2_ to O_2_ by fluorination^25,26^ or by direct water electrolysis^27^. In practice, however, ^17^O was never used due to these complicated measurements.

For a long time, IRMS has been the technique for DLW water analysis. As IRMS functions with pure gases, pre-treatment for the (water) samples is necessary, preceded by distillation if the body fluid is blood^2,4,9,28^. Optical spectroscopic measurement of water vapour has become a reliable alternative, initially thanks to the pioneering activities in our laboratory^14,29,30^. At present, there is commercial equipment available, which enables the measurement of the isotope ratios for the DLW method faster and easier, but with equivalent precision and accuracy compared with IRMS^20,31–34^. The advantage of optical spectrometry is that all water isotopologues can be measured simultaneously, so the analysis of ^2^H, ^18^O and ^17^O of water samples and biological fluids is possible in one sample measurement. This provides the possibility to add this third isotope, ^17^O, to DLW analysis, and turn it into Triply Labelled Water (TLW). Of course, then also water highly enriched in all three rare isotopes should be administered to the study subjects. At the moment, due to almost absent demand, water enriched in both ^18^O and ^17^O water is a non-standard product, but that might change when the use of TLW will become more widespread.

In this study, we make use of this possibility, and demonstrate, to our knowledge for the first time, complete TLW measurements for the analysis of the CO_2_ production of mice in different diet types. Triply highly enriched ^2^H, ^17^O and ^18^O water was injected into mice, and isotope enrichment of the distilled blood samples were measured by optical spectrometry, using available reference waters of ^2^H and ^18^O, and home-made ^17^O reference waters. We describe how we conduct the TLW method and give several calculation protocols. Then we analyze the advantage of the TLW method, the difference of calculation protocols, the deviation of CO_2_ production measured by TLW and indirect calorimetry, and the influence of different nutrition for mice. The last step, converting the produced CO_2_ to energy expenditure, is a mere multiplication by the energy equivalent value for the food. Since this study focusses on method evaluation, we refrain from this step and stick with the produced CO_2_.

Giving this study an additional goal, we chose to repeat a previous experiment by our group^13^, in which we compared the energy expenditure measured by DLW to Indirect Calorimetry (IC), for mice on low fat (LF) and High Fat (HF) diets. That experiment showed systematic differences between DLW and IC results, and by its repetition, now using TLW, we wanted to find out if this difference occurred again, and if so, if the addition of ^17^O would shed light on this difference.

## Material and methods

### Animals and housing

Twenty male C57BL6/J mice were individually housed on a 12:12 light–dark cycle with food and water ad libitum, and a controlled temperature (22 ± 1 °C) (more details in Ref.^13^). At the age of 27 weeks, ten of the mice were maintained on regular chow diet, the so-called low-fat diet (LF) group (17.5 kJ/g; fat content 13.5%; protein content 28%; carbohydrate content 58%). The other ten mice were changed to a high-fat sucrose diet (HF) (21.8 kJ/g; fat content 28%; protein content 19.5%; carbohydrate content 52.5%) eleven weeks prior to the TLW injection.

### Preparation of the triply labelled water

We produced a highly enriched TLW mixture by mixing the ^2^H, ^18^O and ^17^O “mother” waters (around 8.0, 12.4 and 6.2 g, respectively, determined with 0.1 mg precision). The “mother” waters are purely ^2^H water ([^2^H] > 99.9%, Sigma-Aldrich, Netherlands), ^18^O water ([^18^O] ≈ 98%, Rotem industries, Rehovoth, Israel) and ^17^O water with high enrichment levels ([^17^O] ≈ 41%, [^18^O] ≈ 43%, Rotem industries, Rehovoth, Israel). This resulted in a mixture ([^2^H] = 29.7%, [^18^O] = 55.88%, [^17^O] = 8.55%). This is equivalent to enrichment factors of ≈ 1900, 280 and 225, respectively, so in our experiments we expect higher enrichments for δ^2^H than for δ^18^O and δ^17^O, whereas the latter two will be roughly equal. Given the measurement uncertainty of our Off-Axis Integrated Cavity Output Spectroscopy (OA-ICOS) analyzer, we expect similar accuracies in the measurements for all three isotopes this way.

Using this mixture for the injection in the mice, we estimated that a 0.17 g injection would result in the initial samples (the most enriched ones) having δ^2^H and δ^18^O values close to the international enriched reference water IAEA-609 (δ^2^H = 16,036.4‰, δ^18^O = 1963.7‰^35^). Therefore, the suite of enriched water references IAEA-609,608 and 607 are suitable for δ^2^H and δ^18^O calibration of all mice blood samples. However, as these reference waters are not (or only mildly) enriched in ^17^O, we cannot use them for the calibration of our δ^17^O measurements, where we expect initial values of around 1700‰. Therefore, to calibrate TLW measurements, we made a range of four reference waters enriched in all three isotopes, by gravimetrically mixing the highly enriched TLW mixture ([^2^H] = 29.7%, [^18^O] = 55.88%, [^17^O] = 8.55%) with varying quantities of demineralized tap water (δ^2^H =  − 42.49‰, δ^18^O =  − 6.36‰, δ^17^O =  − 3.39‰). Different amounts of the TLW mixture, from 0.25 to 0.6 g (~ 0.1 mg precision), were put into a 2 ml glass vial, and then immersed into a glass bottle which contains about 100 g demineralized water (~ 0.1 mg precision). These bottles were sealed after mixing and shaken periodically for several hours.

Table 1 shows the values of these four TLW-references along with their uncertainty. The δ^2^H and δ^18^O values were measured by OA-ICOS and calibrated using IAEA-609, 608 and 607. The error of δ^2^H is based on several measurement repetitions, but for δ^18^O, the measurement uncertainty is small (less than 1‰), so the uncertainty of IAEA waters are important for the δ^18^O error of these TLW-references^35^.

**Table 1:** Isotope δ-values of the enriched TLW reference waters.

Enriched TLW reference water	δ^2^H (‰)	Measured error (‰)	δ^18^O (‰)	Measured error (‰)	δ^17^O (‰)	Fitted error (‰)
REF-1	13,714	12	2009.2	2.3	1630	16
REF-2	11,079	9	1619.9	2.3	1300	13
REF-3	8400	9	1225.2	2.2	1000	10
REF-4	4370	8	636.0	1.0	520	5

The δ^2^H and δ^18^O values were measured by OA-ICOS and calibrated using the IAEA-reference waters. The δ^17^O values are based on several dilution experiments, with a relative uncertainty of ± 1% attributed to them.

The IAEA waters are unfortunately only mildly enriched in ^17^O, so for the δ^17^O value determination of our four TLW reference waters, we conducted several dilution experiments to bring the resulting δ^17^O values of the diluted TLW reference waters within the range of the IAEA waters (with IAEA-609 having the maximum δ^17^O value of 126.6‰). By using the accurately determined dilution factor, we could in this way calibrate the δ^17^O of TLW reference waters using IAEA-609,608 and 607. All calculations of isotope abundances and δ-values were performed using a thoroughly validated Excel spreadsheet^36^. Based on the measurement uncertainties, the uncertainties in the values quoted for the IAEA waters, and the dilution uncertainties we attribute a conservative ± 1% relative uncertainty to our δ^17^O values (see Table 1). Besides the best estimates for the δ^17^O of each TLW reference water, we also could determine the abundances of the highly enriched TLW ([^2^H] = 29.7%, [^18^O] = 55.88%, [^17^O] = 8.55%). We separately determined that the ^18^O mother water has a 98.42% abundance, deviating somewhat from 98.2% provided by the manufacturer (but within their specification). The ^17^O mother water contains 34.47% [^17^O] and 42.65% [^18^O] (for [^17^O] deviating from the manufacturer's specification of 41.1% whereas [^18^O] with 43% agrees). The ^2^H mother water is virtually pure. The enriched reference waters and the highly enriched TLW mixtures were stored in thoroughly closed bottles inside a sealed container, filled with dry N_2_ gas at slight overpressure. This prevented the uptake of—and thus dilution by—atmospheric moisture.

### Experimental design

For the experiments, each mouse was intraperitoneally injected with about 0.17 g (weighted to the nearest 0.1 mg) of the highly enriched TLW mixture. Before injection, we took 4 blood samples separately from 5 mice for background isotope analysis. After exactly 2 h after the TLW injection, the initial blood samples were taken. Then, the mice were transferred to the indirect calorimetry (IC) cages for two consecutive days. The indirect calorimetry (IC) was shortly interrupted at exactly (deviations less than 2 min) 24 and 48 h after the initial sample time. In this study, the background and TLW mixture blood were all sampled by tail snip (4 times per sample every time), and then flame-sealed into 25 µl glass capillaries until the micro-distillation process^13^. Mice body masses were measured by a balance (~ 0.1 g precision), and fat, lean weight and water content of all the mice were measured by a magnetic resonance imaging machine (EchoMRI-100, Echo Medical Systems, Houston, TX, US) just before injection^37^. All experimental procedures were approved and guided by the local Animal Experimentation Committee (DEC) of the University of Groningen.

### Indirect calorimetry

In the IC cages (Homemade polypropylene cage, 43 × 30 × 21 cm), the housing and feeding conditions were not changed. The detailed description of the IC method in our lab is in Ref.^13^. The IC system measured the O_2_ and CO_2_ concentration difference of the dried inlet air (3 Å molecular sieve drying beads; Merck, Darmstadt, Germany) and dried outlet air going through the chambers. The flow rate of the inlet was set at 20 l/h, and only 6 l/h outlet air passed through the drying system and subsequently to the gas analyzers. The mass-flow controllers (Type 5850; Brooks Instrument, Veenendaal, The Netherlands) were calibrated before and after the trials (the variation < 1%). O_2_ was measured by a paramagnetic O_2_ analyzer (Xentra 4100, Servomex, Egham, UK), and CO_2_ by an infrared gas analyzer (Servomex 1440). The CO_2_ and O_2_ analyzers were calibrated daily with two certified gas standards (Linde Gas, The Netherlands), with values 19.5612% for O_2_ and 0.0006% for CO_2_, and 20.8743% for O_2_ and 0.5133% for CO_2_, respectively. The maximum overall error of the method is ≤ 2%. For validation purposes, the respiratory quotients (RQ = r_CO2_/r_O2_) and metabolic rates (MR) were also recorded and calculated^13,38^.

### Analysis method of the TLW samples

The δ^2^H, δ^17^O and δ^18^O of mice blood samples and reference waters were measured by a commercial Off-Axis Integrated Cavity Output Spectroscopy (OA-ICOS) Liquid Water Isotope Analyser (LWIA 912-0050, Los Gatos Research, San Jose, CA, USA). Before injection into the analyser, all the samples and references were prepared by a home-built micro-distillation system (detailed distillation procedures are described in Ref.^39^). In brief, a capillary is broken in an evacuated system, and the water is collected in a freeze finger immersed in liquid nitrogen. The system is then again evacuated, and the water is finally transferred, again using liquid nitrogen, into a small insert tube, which can be measured directly on the OA-ICOS analyser. The reference waters (the IAEA series and our TLW references) are treated identically, so also transferred from capillaries.

The distilled samples and references were introduced into the OA-ICOS instrument through an auto-injector (Pal, CTC Analytics, Zwingen, Switzerland), and there is a heated injector block to evaporate the liquid water. This vapour expands into a high-finesse optical cavity, and the δ^2^H, δ^18^O and δ^17^O values were calculated from fits to the relative transmission spectrum. The distilled IAEA-609,608 and 607 and our local TLW references are interleaved with samples during the measurement series for calibration and instrumental drift correction. Each reference and sample water was injected 12 times. Before each distillate reference, the same reference water, but without distillation, is also injected 12 times to check the micro-distillation quality and stability, and also to reduce memory effects. However, only the distilled references were used for calibration.

**Figure 1:**
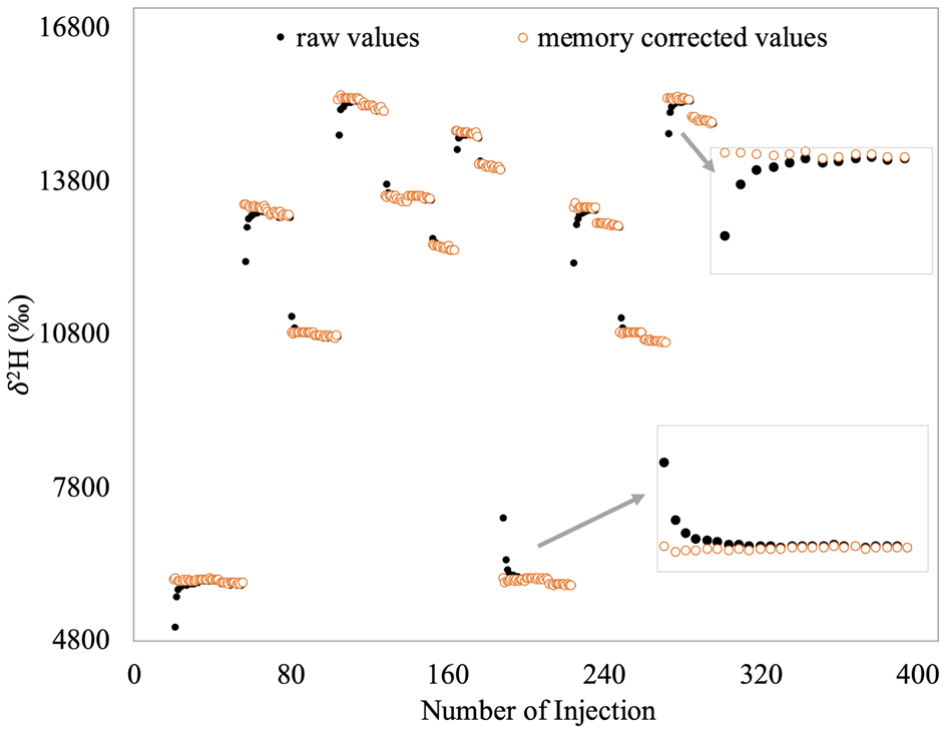
The memory effect of δ^2^H observed in an initial samples’ measurement batch. The measured (raw) values are showed by the black circles and the open red circles indicate the values after memory effect correction by the 3 pools exchange algorithm. In addition to the memory, there is also some drift visible which is also corrected by the data analysis program.

Raw data from the instrument were analysed using a bespoke data analysis program (written in R), through which memory effects and drifts were corrected, and calibration was performed (for details see^40^). Specifically, to correct for memory effects we do not ignore the first few injections of a sample, but instead use all of them and correct for the memory effects using a 2–3 pool exchange algorithm^39^. This is quite meaningful for TLW blood samples, which have a minimal sample size (less than 15 µl). Figure 1 shows a representative part of a measurement batch (containing “initial” samples), in which both the raw, and memory corrected values for several samples and reference waters are shown. The improvement in precision is remarkable: standard deviations of the 12 δ^2^H measurements of the samples around 6000‰, for example, reduce from 210 to 30‰ when measured just after natural (demineralized Groningen tap-) water (δ^2^H ≈ − 42‰).

For calibration, mostly a “multiple-point” quadratic fit is chosen, which is based on three or more of the reference waters. This is based on our experience that for these highly enriched samples and the large range in δ-values in each series (e.g.: δ^18^O from 736 to 1963‰ for the IAEA reference waters), the instrument’s output is not fully linear. This is probably due to imperfect line fitting, which also makes itself noticeable through relatively high values for the so-called “Narrow Band” spectroscopic interference^41^. This tool is meant to be an indicator for contamination, but as contamination does not occur in our samples (and certainly not in the pure reference waters), here it is the result of an imperfection in the spectroscopic fit of these triply labelled waters.

As illustrated in Ref.^39^, duplicate analysis of DLW samples is necessary and helpful, due to the dominant uncertainty contribution of the actual procedure of flame-sealing and micro-distillation. In this study, if the δ^2^H value of a duplicate analysis deviates more than 2% of its value from the first (or 1.5% for δ^18^O, 1.5% for δ^17^O), a third sample is analyzed. A third sample is also taken if the quality of a capillary is questionable (for example not tight or containing too much air). The average of the duplicate (or triplicate if an outlier cannot be identified) analyses, along with the standard error in the mean is taken as the final result. The OA-ICOS measurement uncertainty for individual samples is usually negligibly small compared to the spread between duplicate samples.

For the TLW method analysis, we use isotope abundances instead of the δ-values. First, the sample’s ^x^δ_s_ values need to be converted into abundance ratios ^x^R_s_ (x = 2, 17 and 18), using the isotope abundance ratios for Vienna Standard Mean Ocean Water (VSMOW), which are 1.5576 × 10^–4^, 3.799 × 10^–4^, and 2.0052 × 10^–3^ for ^2^H, ^17^O and ^18^O, respectively^42^:

1 $$\begin{array}{c}{}^{x}{R}_{s}={ }^{x}{R}_{VSMOW}\times \left(1 {+ }^{x}{\delta }_{s}\right).\end{array}$$

From these ratios, the absolute isotope concentrations ^x^C_s_ are computed, usually expressed in parts per million (ppm):

2 $$\begin{array}{c}{}^{2}{C}_{s}=\frac{{ }^{2}{R}_{s}}{1+{ }^{2}{R}_{s}},\end{array}$$

3 $$\begin{array}{c}{}^{18}{C}_{s}=\frac{{ }^{18}{R}_{s}}{1+{ }^{18}{R}_{s}+{ }^{17}{R}_{s}},\end{array}$$

4 $$\begin{array}{c}{}^{17}{C}_{s}=\frac{{ }^{17}{R}_{s}}{1+{ }^{18}{R}_{s}+{ }^{17}{R}_{s}}.\end{array}$$

### Calculations

After the injection of the enriched TLW mixtures into the mice, the enriched rare isotopes are gradually exchanged with the surroundings, and the turnover rate (k; h^−1^) describing the rare isotope concentration decrease can be expressed as:

5 $$\begin{array}{c}{k}_{t}=\frac{ln\left[\frac{\left({C}_{i}-{C}_{b}\right)}{\left({C}_{f}-{C}_{b}\right)}\right]}{t}.\end{array}$$

C is the concentration of the isotope ^2^H, ^18^O or ^17^O. “i” means the initial, and in this study, the initial sample is the 2-h samples taken after injection. “b” is background (concentrations corresponding to δ^2^H =  − 27.3‰, δ^18^O =  − 4.85‰, δ^17^O =  − 2.61‰, as established based on sampling five mice prior to the injection of TLW), and “f” is the the final sample (taken either 24 h or 48 h after the initial sample), therefore, time duration “t” is equal to 24 or 48 h.

The dilution space of the isotopes in the body, and thus the size of the body water pool, can be calculated using the measurement of the initial concentration by the so-called plateau method^4^:

6 $$\begin{array}{c}N{=Mol}_{inj}\frac{{C}_{i}-{C}_{inj}}{{C}_{b}-{C}_{i}},\end{array}$$

where N (mol) represents the dilution space or body water pool for ^2^H (N_2H_), ^18^O (N_18O_) and ^17^O (N_17O_). Mol_inj_ is the number of the moles of the injection TLW (19.81 g/mol) and C_inj_ is the injected enrichment ([^2^H] = 29.7%, [^18^O] = 55.88%, [^17^O] = 8.55%). In this expression, the loss of enriched isotopes in the 2 h between the injection and the initial measurement is ignored. Alternatively, one can take this loss into account by extrapolating the turnover rate back to the injection time. This is called the intercept method^4^:

7 $$\begin{array}{c}N{=Mol}_{inj}\frac{{C}_{i-ic}-{C}_{inj}}{{C}_{b}-{C}_{i-ic}},\end{array}$$

8 $$\begin{array}{c}{C}_{i-ic}=\left({C}_{i}-{C}_{b}\right){e}^{{k}_{i}\left({t}_{i}-{t}_{inj}\right)}+{C}_{b},\end{array}$$

where C_i–ic_ is the concentration extrapolated back to the time of injection (0 h), and t_i_–t_inj_ is equal to 2 h in our case.

Whereas the plateau method is expected to underestimate the body water pool slightly (as the loss of enriched isotopes during the first 2 h is ignored), the intercept method, on the other hand, possibly overestimates the body water pool, as the loss of enriched isotopes during the first two hours is probably less than later, since the enriched isotopes have not distributed themselves over the entire body water pool. Therefore, calculating and comparing both is a good practice.

It is generally observed that the body water pool as determined by ^2^H is slightly, but significantly, larger than that by ^18^O^4,13,43^. This is commonly attributed to the exchange of hydrogen (and thus ^2^H) with body tissues, which does not occur with oxygen. For this reason, we expect the body water pool determination using ^17^O to be identical to that with ^18^O.

The amount of total body water (TBW, g) for each individual animal is then simply:

9 $$\begin{array}{c}TBW=M\times N.\end{array}$$

M is the molar mass of water (18.02 g/mol). In terms of carbon dioxide production, in a simple expression ignoring the fractionation effects, the difference between ^2^H and ^18^O turnover is proportional to the rate of CO_2_ production (rCO_2_; mol/h):

10 $$\begin{array}{c}{r}_{CO2}=\frac{N}{2}\times \left({k}_{18\text{O}}{-k}_{2\text{H}}\right).\end{array}$$

Also here, several fractionation effects occur in the process. Therefore, this Eq. (10) is not suitable for an accurate calculation of rCO_2_. However, as the deviations are relatively small, this equation can be used for uncertainty propagation calculations. The full expression contains the following fractionation factors: the (partly kinetic, partly equilibrium) evaporation of water for ^2^H (f_1_) and ^18^O (f_2,18O_), and the CO_2_-H_2_O fractionation for ^18^O (f_3,18O_), which is assumed to be in equilibrium:

11 $$\begin{array}{c}{r}_{CO2}=\frac{N}{2\times {f}_{\text{3,18}}}\times \left({k}_{18\text{O}}-{k}_{2\text{H}}\right)-{r}_{G}\times \frac{{f}_{\text{2,18O}}-{f}_{1}}{2\times {f}_{\text{3,18O}}}\times N\times {k}_{2\text{H}}.\end{array}$$

r_G_ is the fraction of the water loss due to evaporation, as it happens in the lungs. By lack of a firm determination or estimate, most studies use a value of 0.5. The isotopic fractionation process leads to relatively lower abundances of the heavy isotopes in the vapour phase. All fractionation factors are shown in Table 2, and Eq. (11) is from Ref.^4^. If instead of on ^18^O and ^2^H turnover, rCO_2_ is calculated based on the ^17^O and ^2^H turnover, we arrive at the identical equation, but with the ^17^O decay rate, and two fractionation factors now for ^17^O:

12 $$\begin{array}{c}{r^{\prime}}_{CO2}=\frac{N}{2\times {f}_{\text{3,17O}}}\times \left({k}_{17\text{O}}-{k}_{2\text{H}}\right)-{r}_{G}\times \frac{{f}_{\text{2,17O}}-{f}_{1}}{2\times {f}_{\text{3,17O}}}\times N\times {k}_{2\text{H}},\end{array}$$

where k_17_ is the turnover rate for ^17^O, and f_2,17O_ and f_3,17O_ are the fractionation factors for ^17^O fractionation in the water evaporation and the CO_2_-H_2_O equilibrium, respectively.

**Table 2:** Isotope fractionation factors measured in vitro for the equilibrium and kinetic exchanges of ^2^H, ^18^O and ^17^O between liquid water and vapor, and for the ^18^O, ^17^O between water and gaseous CO_2_ at 37 °C^4,15,17–19,25^.

Fractionation factor	Equilibrium	Kinetic	Final factors used in this study from equilibrium: kinetic = 3:1
f_1_	0.941	0.9235	0.9366
f_2,18O_	0.9925	0.976	0.9884
f_3,18O_	1.0389		
f_2,17O_	0.996	0.9872	0.9938
f_3,17O_	1.0202		
(f_2,18O_–f_1_)/2f_3,18O_			0.0249
(f_2,17O_–f_1_)/2f_3,17O_			0.0280

In Table 2, we list the fractionation factors obtained from literature, as well as the 'mixed' results by the equilibrium/kinetic as a ratio of 3:1^4^, and the final (f_2_–f_1_)/2f_3_ calculation results. All the factors are equal to the values listed in Refs.^4,16^, expect the f_3,17_. Its value of 1.0202 is obtained based on the equation from^17^ at 37 °C, and ln(α17)/ln(α18) = 0.5229^44^.

There are several classical equations to calculate the CO_2_ production, which differ in the selection of fractionation factor values, portion of fractionation water (r_G_) and body water pool models, and are also dependent on the research subjects (animals or humans)^4,16,45–47^. Equations (11) and (12) use a single pool model, they are reproduced as equationns 1-1 and 1-2 in Table 3. For ^18^O, equation 1-1, is similar to the expression in Ref.^4^ except the number of decimal places, and equation 1-2 is for ^17^O based on the same calculation principle. When we consider the two-pools model, which means that the effective body water pool is taken differently for ^2^H than for ^18^O (or ^17^O), the Coward 1985^46^ and Speakman 1993^45^ models are more logical and suitable for animal CO_2_ production calculation. The equations 2-1, 2-2, 3-1 and 3-2 in Table 3 are based on their model principle, separately from Coward 1985^46^ and Speakman 1993^45^. The fractionation factors (from Table 2) used for the equations in Table 3 are the same, irrespective of the model. The R_dil_ in 3-1 and 3-2 is the mean dilution space ratio N_2H_/N_O_ (the dilution space calculated by ^2^H divided by the dilution space calculated by ^17^O or ^18^O) for different group members, so different for the low and high fat diet mice.

**Table 3:** CO_2_ production (r_CO2_) calculation equations by the doubly labelled water method separately based on ^18^O or ^17^O with ^2^H^4,45,46^.

No.	Author	pools	Equation	N
1-1	Speakman Book 1997	1	${r}_{CO2}=\frac{N}{2.0778}\left({k}_{18\text{O}}-{k}_{2\text{H}}\right)-{r}_{G}\times 0.0249N{k}_{2\text{H}}$	N = N_18O_
1-2			${r^{\prime}}_{CO2}=\frac{N}{2.0404}\left({k}_{17\text{O}}-{k}_{2\text{H}}\right)-{r}_{G}\times 0.028N{k}_{2\text{H}}$	N = N_17O_
2-1	Coward 1985	2	${r}_{CO2}\text{=}\frac{1}{2.0778}\left({{N}_{18\text{O}}k}_{18}-{{N}_{2\text{H}}k}_{2\text{H}}\right)-{r}_{G}\times 0.0249{N}_{2\text{H}}{k}_{2\text{H}}$	
2-2			${r^{\prime}}_{CO2}=\frac{1}{2.0404}\left({{N}_{17\text{O}}k}_{17\text{O}}-{{N}_{2\text{H}}k}_{2\text{H}}\right)-{r}_{G}\times 0.028{N}_{2\text{H}}{k}_{2\text{H}}$	
3-1	Speakman 1993	2	${r}_{CO2}=\frac{N}{2.0778}\left({k}_{18\text{O}}-{R}_{dil}{k}_{2\text{H}}\right)-{r}_{G}\times 0.0249N{R}_{dil}{k}_{2\text{H}}$	$N=\frac{\left({N}_{18\text{O}}+\frac{{N}_{2\text{H}}}{{R}_{dil}}\right)}{2}$
3-2			${r^{\prime}}_{CO2}=\frac{N}{2.0404}\left({k}_{17\text{O}}-{R}_{dil}{k}_{2\text{H}}\right)-{r}_{G}\times 0.028N{R}_{dil}{k}_{2\text{H}}$	$N=\frac{\left({N}_{17\text{O}}+\frac{{N}_{2\text{H}}}{{R}_{dil}}\right)}{2}$

### Ethics approval

All experimental procedures involving animals were approved and guided by the local Animal Experimentation Committee (DEC) of the University of Groningen.

### Consent for publication

All authors whose names appear on the submission consent to this version of the manuscript to be published.

## Results

### Body composition

After 11 weeks on a high-fat diet, the high-fat diet mice gained more than 5 g of weight. On the basis of the body mass gain (> 10 g or < 10 g), 5 mice were assigned to be obesity-resistant (HF-OR), and 5 mice were in the obesity-prone (HF-OP) group. The body mass weighed just before injection were used as body weight, together with the fat mass, lean mass, and body water measurement by EchoMRI. From the 10 mice on low fat diet (LF), two had to be discarded from the data set due to blood sampling problems (only one successful capillary for the initial sampling, and large discrepancies between their calculated total body water by TLW and by EchoMRI).

**Figure 2:**
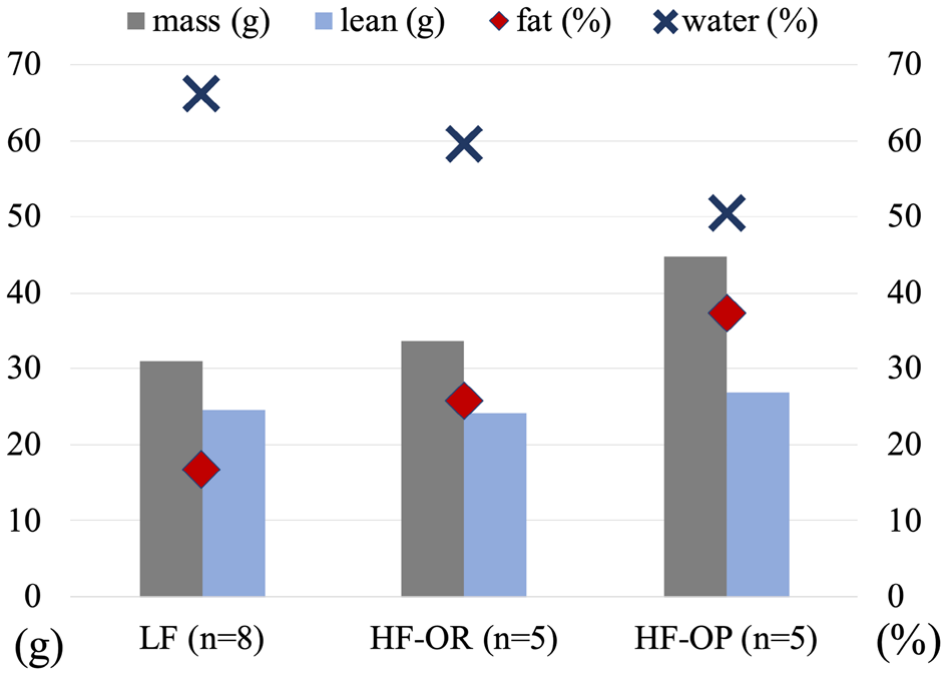
The average body-weight (g), fat percentage, body-water percentage and lean content (g) for the low fat (LF) mice group, high fat obesity-resistant (HF-OR) mice group and high fat obesity-prone (HF-OP) mice group.

Figure 2 illustrates the average values of the body-weight, lean content (g), fat percentage (fat/body-weight) and body water percentage (body-water/body-weight) of the three mice groups: LF, HF-OR and HF-OP (with 8, 5 and 5 individuals, respectively). When analyzing the individual differences in body content, we find that the fat percentage is positively correlated with the body-weight, and negatively correlated with the body-water percentage. When analyzing the group difference, Fig. 2 clearly shows that the HF-OP mice group, which is heaviest (the average 45 g), has the lowest water percentage (the average 50%) and the highest fat percentage (37%). The average weight difference between the LF and HF-OR groups is not large (30.6 g and 33.8 g, respectively), but the fat-% and water-% are quite different. The lean contents for LF and HF-OR groups are similar (nearly 24 g), and the average lean content of the HF-OP group is only 2.5 g higher than the other two groups.

### Indirect calorimetry

After taking the initial blood samples, the 20 mice were put into the indirect calorimetry box and the actual r_CO2_, r_O2_ and RQ were measured over the two following days. The data around the interruptions (taking blood samples) were removed, and the IC data summary of the two days is listed in Table 4. Table 4 also shows the mean food intake (kJ/day) for the three categories. The individual variability is large (especially for the HF-fed mice), but on average the LF mice have taken in a higher caloric value of food than the HF ones.

**Table 4:** Indirect calorimetry (IC) measurement results for 2 days, expressed as mean ± standard error (SE).

	Time	LF (n = 8)	HF-OR (n = 5)	HF-OP (n = 5)
rCO_2_ (ml/h)	Day 1	77.8 ± 2.1	83 ± 4	91.4 ± 2.0
	Day 2	83.4 ± 1.4	83.0 ± 2.4	91 ± 3
rO_2_ (ml/h)	Day 1	99.1 ± 2.3	118 ± 5	128 ± 4
	Day 2	100.5 ± 1.9	113 ± 4	124 ± 3
RQ (CO_2_/O_2_)	Day 1	0.785 ± 0.017	0.705 ± 0.008	0.713 ± 0.009
	Day 2	0.828 ± 0.006	0.736 ± 0.008	0.730 ± 0.008
MR (kJ/day)	Day 1	47.5 ± 0.9	55.0 ± 2.2	59.8 ± 1.6
	Day 2	48.4 ± 0.7	53.0 ± 1.7	58.3 ± 1.6
food intake (kJ/day)	Day 1	46 ± 5	20 ± 7	36 ± 12
	Day 2	47 ± 3	33 ± 8	35 ± 3

Day 1 means the first 24 h in the IC box after the initial samples taken, and Day 2 is the second 24 h. rCO_2_ and rO_2_ are the CO_2_ production and O_2_ consumption in ml per hour, RQ is the respiratory quotient. The metabolic rates (MR) are calculated based on Ref.^38^. The lowest rows give the mean food intake (kJ) ± SE for the three categories.

All observed values are equal within the error for day 1 and day 2, except for r_CO2_ of the LF mice. The r_CO2_ of the LF and HF-OR groups are similar and their difference is within the error, but the CO_2_ production of the HF-OP group is about 8 ml/h more than that of the other two groups. The r_O2_ values differ significantly between the groups, and that of the HF-OP group is the highest.

The respiratory quotient (RQ) resembles the low-fat or high-fat food intake difference between the LF and the two HF groups. There is no difference between the HF-OR and HF-OP groups, and they are both lower than the RQ values of LF. As the Metabolic Rate values are directly computed from r_CO2_ and the RQ, they show the same trends. The MR value of HF-OP is highest (about 5 kJ/day more than HF-OR, 11 kJ/day more than LF).

### Turnover rates k

**Figure 3:**
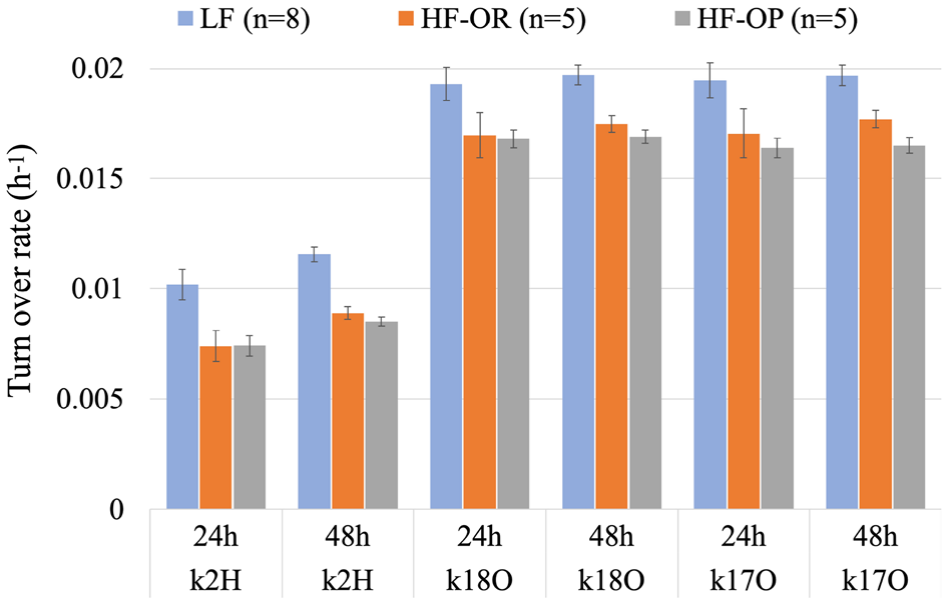
The average turnover rates (h^−1^) of ^2^H (k_2H_), ^18^O (k_18O_) and ^17^O(k_17O_) for the LF (blue, n = 8), HF-OR (red, n = 5), and HF-OP (grey, n = 5) mice groups based on the final samples taken at 24 h or 48 h after taking the initials. The error bar is ± SE.

The turnover rates for ^2^H (k_2H_), ^18^O (k_18O_) and ^17^O (k_17O_) were separately calculated from the logarithmic decline of the initial isotope abundance (2-h after injection) and the isotope abundance of two finals (24 h and 48 h after the initial sample taken). The average k_2H_, k_18O_, k_17O_ for the three mice groups are shown in Fig. 3, whereas Table 5 gives the numerical values, and in addition the turnover rate ratios and differences. All are presented for the 24 h and the 48 h period. It is clear that the LF group has the highest turnover rates of the three groups, and the differences are highly significant between LF and HF groups. The turnover rates of the HF-OR group are slightly higher than the HF-OP group, but for k_2H_ and k_18O_, the difference is not significant.

**Table 5:** The turnover rates (10^−3^ h^−1^) for ^2^H (k_2H_), ^18^O (k_18O_) ^17^O (k_17O_); turnover rates ratio: k_18O_/k_2H_ and k_17O_/k_2H_; turnover rates differences (10^−3^ h^−1^): k_18O_–k_2H_ and k_17O_–k_2H_.

Mice group	k_2H_ (10^–3^ h^−1^)	k_18O_ (10^–3^ h^−1^)	k_17O_ (10^–3^ h^−1^)	k_18O_/k_2H_	k_17O_/k_2H_	k_18O_–k_2H_ (10^–3^ h^−1^)	k_17O_–k_2H_ (10^–3^ h^−1^)
**24-h-final**							
LF (n = 8)	10.2 ± 0.7	19.3 ± 0.8	19.5 ± 0.8	1.93 ± 0.07	1.94 ± 0.07	9.13 ± 0.19	9.3 ± 0.3
HF-OR (n = 5)	7.4 ± 0.7	17.0 ± 1.0	17.1 ± 1.1	2.33 ± 0.12	2.34 ± 0.11	9.6 ± 0.4	9.6 ± 0.5
HF-OP (n = 5)	7.4 ± 0.5	16.8 ± 0.4	16.4 ± 0.4	2.28 ± 0.08	2.23 ± 0.10	9.38 ± 0.16	9.0 ± 0.3
**48-h-final**							
LF (n = 8)	11.6 ± 0.4	19.7 ± 0.4	19.7 ± 0.5	1.710 ± 0.018	1.71 ± 0.021	8.16 ± 0.13	8.15 ± 0.17
HF-OR (n = 5)	8.9 ± 0.3	17.5 ± 0.4	17.7 ± 0.4	1.97 ± 0.05	1.99 ± 0.04	8.6 ± 0.3	8.79 ± 0.23
HF-OP (n = 5)	8.54 ± 0.21	16.9 ± 0.3	16.5 ± 0.4	1.98 ± 0.03	1.93 ± 0.03	8.37 ± 0.16	7.97 ± 0.22

The ratios and differences are calculated from the individual initial isotope abundances (2-h after injection) and two finals (24 h and 48 h after the initial sample taken), and are expressed as mean ± SE.

In Table 5, it is clear that the uncertainty in k-48 h is lower than that in k-24 h, this is because of the larger difference between the isotope values for the 48 h-finals and the initials. In Table 5 and Fig. 3, for k_2H_, we can see the difference between 24- and 48 h is significant, k_2_-48 h being higher than k_2H_-24 h for all three mice groups. For k_18O_ and k_17O_, on the other hand, the differences between the turnover rates for 24 h and 48 h are small and not significant. The k_17O_ and k_18O_ agree with each other within the uncertainties for both of the two times. This is to be expected, as both the ^17^O and ^18^O label are subject to the same processes. The fact that they do agree within the uncertainty increases the confidence in the experimental results (both the animal handling side and the isotopic analysis of the blood samples). Therefore, it is possible to obtain the turnover of oxygen by taking the average of k_17O_ and k_18O_, which lowers the uncertainty of k_O_ (turnover for oxygen isotopes).

As shown in Table 5, the turnover rates ratios k_O_/k_2H_ are typically 1.9 (LF) and 2.3 (HF) for 24 h finals, while for 48 h, they are a bit lower: 1.7 (LF), 2.0 (HF), fully caused by the increase of k_2H_. In the analysis of k_O_–k_2H_, which carries the r_CO2_ signal information, the k_O_–k_2H_ values are obviously lower for 48 h than for 24 h because of the higher k_2H_-48 h values. On the other hand, the differences between the three groups for the same final are not significant in most of the cases, expect the k_17O_–k_2H_ for HF-OR group at 48 h-final.

### Total body water (TBW) and dilution space

**Figure 4:**
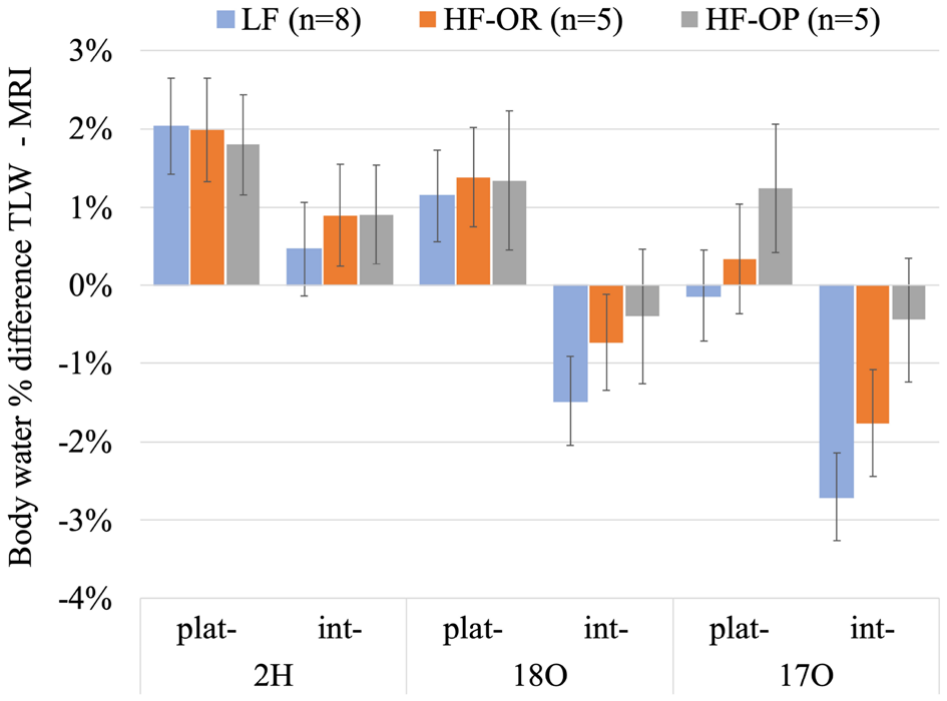
Body water percentage differences between TLW (both ^2^H, ^18^O, and ^17^O) and EchoMRI for the three mice groups, expressed as mean ± SE. Plat—means using the plateau method (Eq. (6)), an int—means using the intercept method (Eq. (7)). The uncertainty for the EchoMRI values is estimated to be 2%.

The body dilution space (N) can be measured based on the plateau method (Eq. (6)) or on the intercept method (Eq. (7)), and the total body water is calculated using Eq. (9). In Fig. 4 we compare the body water percentage (water/mass) results calculated by TLW with those by EchoMRI. To illustrate the differences better, Fig. 4 shows the differences between the two. In terms of individual variation, the water percentage values from the calculation (TLW) and measurement (EchoMRI) are consistent with each other (not shown here). Moreover, the dilution space difference obtained by the intercept method based on the k-24 h or k-48 h is not significant (less than 0.1%), so we only illustrate the percentage difference of the plateau and 48 h-intercept method in Fig. 4.

It is clear in Fig. 4, and expected (see above), that the plateau method gives higher water percentages than the intercept method for both ^2^H, ^18^O, and ^17^O, and also that the water percentages based on ^2^H are the highest (nearly 2% higher than the EchoMRI values for the plateau method). On the other hand, the intercept method results for ^18^O and ^17^O give lower water percentages than that of EchoMRI. Still, given the combination of the indicated uncertainties for the TLW method (as indicated in Fig. 4) and the uncertainty of the EchoMRI method (estimated to be ≤  ± 2%), all differences shown in Fig. 4 are not significant. Furthermore, although the water percentage values for the three different mice groups are different (see Fig. 2), the differences between the TLW and EchoMRI methods do not seem to correlate with these percentages themselves.

The absolute total body water or dilution space (N) values calculated by three isotopes and two methods are listed in Table 6 for the three mice groups. As Fig. 4 already showed, the values of the plateau method are higher than that of the intercept method for each mice group irrespective of the isotope used. N_2H_ is always higher than N_O_, and N_17O_ matches N_18O_. The most important feature in Table 6 is that the dilution space of HF-OP group is significantly higher than that of LF and HF-OR, while the dilution spaces of HF-OR and LF are equal for most of the cases.

**Table 6:** The dilution space calculated using either ^2^H (N_2H_), ^18^O (N_18O_) and ^17^O (N_17O_) with the plateau or intercept methods for the three mice groups.

Mice group	N_2H_ (mol)		N_18O_ (mol)		N_17O_ (mol)	
	Plateau	Intercept	Plateau	Intercept	Plateau	Intercept
LF (n = 8)	1.17 ± 0.03	1.14 ± 0.03	1.156 ± 0.025	1.111 ± 0.025	1.134 ± 0.022	1.091 ± 0.022
HF-OR (n = 5)	1.153 ± 0.022	1.13 ± 0.03	1.142 ± 0.021	1.102 ± 0.021	1.122 ± 0.023	1.085 ± 0.023
HF-OP (n = 5)	1.30 ± 0.04	1.27 ± 0.04	1.29 ± 0.04	1.24 ± 0.04	1.28 ± 0.04	1.24 ± 0.04

The values are expressed as mean ± SE.

### CO_2_ production

**Figure 5:**
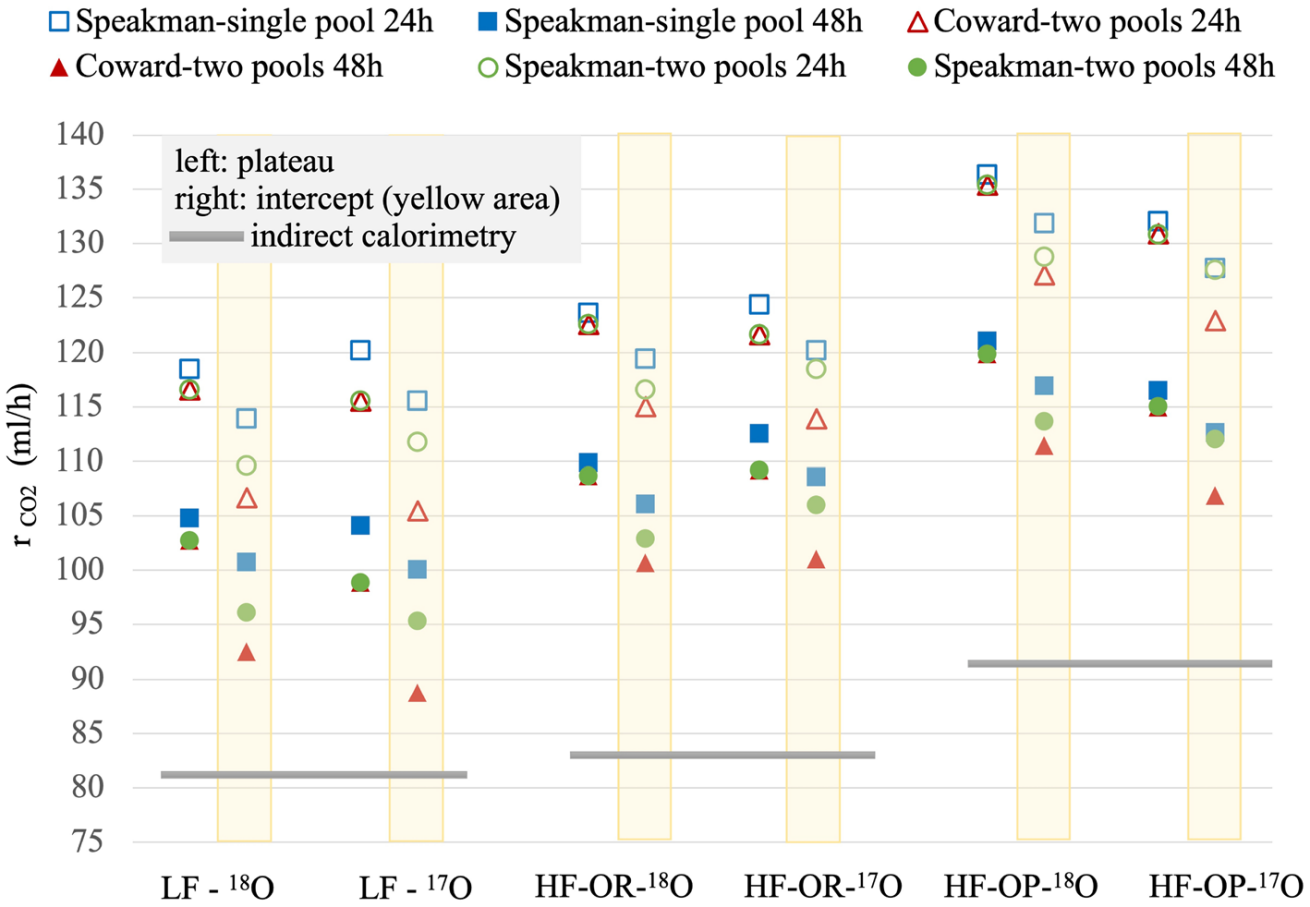
The average r_CO2_ (ml/h) of the three mice groups (LF, HF-OR and HF-OP) based on different calculation protocols. There are three models (Speakman single- or two-pools model, Coward two-pools model), two kinds of doubly labelled water method (^18^O–^2^H, or ^17^O–^2^H), two dilution space calculation methods (plateau or intercept), and two different finals (24 h or 48 h). The grey horizontal lines in the bottom are the average r_CO2_ values of two days from the indirect calorimetry method.

The average r_CO2_ of each LF, HF-OR and HF-OP group are calculated by three kinds of equations which are listed in Table 3. We took the r_G_ = 0.5. The r_CO2_ results are shown in Fig. 5, and for each group, there are two kinds of doubly labelled water methods: left columns based on ^18^O and ^2^H, and right columns based on ^17^O and ^2^H. We also consider the different dilution space (N) calculation methods (plateau or intercept methods) and two different finals (24 h or 48 h). The grey horizontal line in the bottom is the average r_CO2_ value for 2 days from the indirect calorimetry method for the three mice groups. Therefore, in summary, we consider 4 factors for each mice group to calculate the r_CO2_: two finals (24 h or 48 h), two N calculation methods (plateau or intercept), three models (Table 3^4,45,46^), and two isotope combinations (based on ^2^H–^18^O or ^2^H–^17^O). The relative uncertainties of the classical DLW (^18^O and ^2^H) methods in this study are 8.5% (24 h) and 5.1% (48 h), while for (^17^O, ^2^H) DLW they are 6.3% (24 h) and 4.0% (48 h). The relative uncertainty in the indirect calorimetry values is estimated to be ≤  ± 2%.

For all three mice groups, the 24 h r_CO2_ data are much higher than the 48 h data, irrespective of the calculation method, in other words, the differences between solid and hollow symbols in each column are the same. The differences are all caused by the k_2H_-48 h value being larger than the k_2H_-24 h one (see Fig. 3, Table 5). Of course, this difference could indicate a real different behavior, but the IC values for the two days do not show such a difference (Table 4). In terms of different oxygen isotopes for each mice group, the difference is random (from 0 to 8 ml/h), just within the largest errors no matter which model is used. For each of the oxygen isotope and models in each mice group, the r_CO2_ calculated by the intercept method (yellow area) is lower than that by the plateau method. However, the differences are within the uncertainties. Interestingly, the intercept points are more scattered than the plateau ones, caused by the extra influence the turnover rates k have when using the intercept method. As was stated before, one can expect the plateau method to give an overestimation of the water pool size, and the intercept an underestimation. This results in and over- and underestimation of the r_CO2_, respectively. The average of the two values would probably produce the best result, while their differences would give an estimate of the uncertainty.

Obviously, the most striking feature of Fig. 5 is the discrepancy between all the TLW results on the one hand, and the IC results on the other. The discrepancy is the smallest for the 48 h two-pools models, with the solid triangles (Coward 1985, 48 h-) having the lowest discrepancy with IC for each mice group. The lowest difference of TLW and IC is 8 ml/h (LF) and 16 and 15 ml/h (HF-OR and HF-OP) for these results (the equation 2-2 for 48 h). The TLW and IC results agree in the sense that both show the highest r_CO2_ for the HF-OP group. However, the average IC values for the HF-OR and LF groups are similar, while in the TLW methods, the r_CO2_ of HF-OR is higher than the r_CO2_ of the LF group (about 10 ml/h).

**Figure 6:**
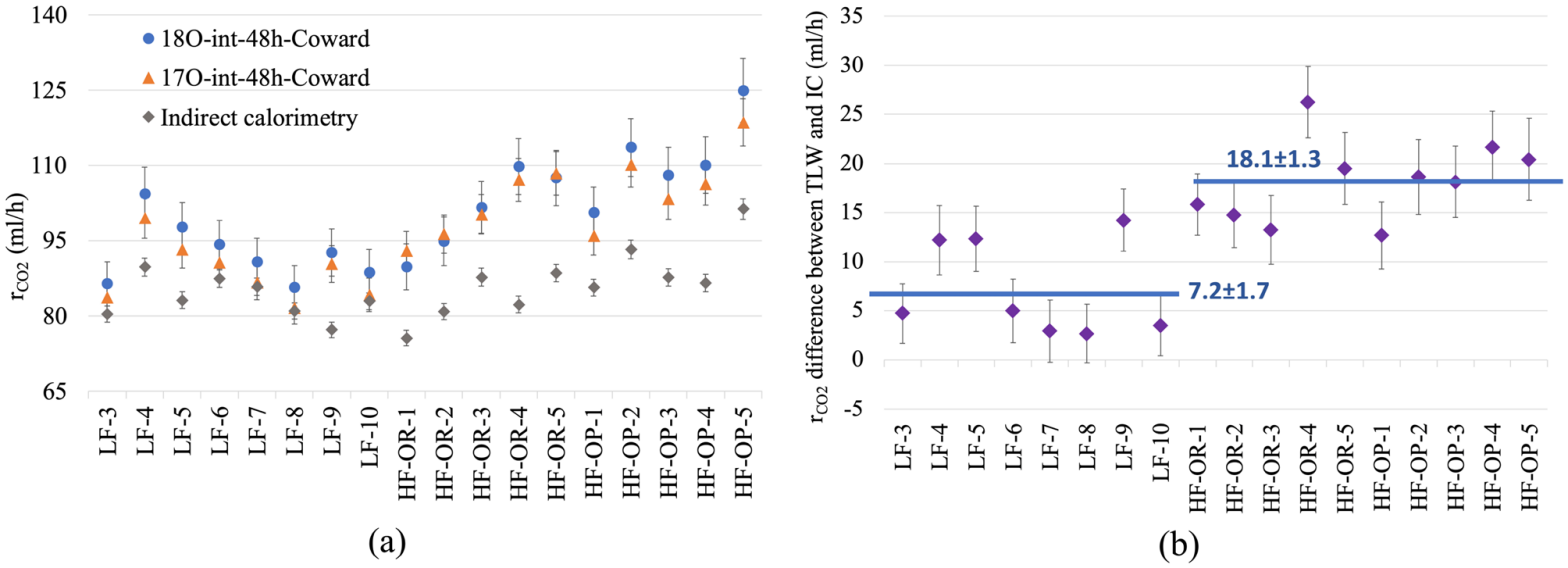
**(a)** Individual r_CO2_ values (ml/h) measured by ^18^O–^2^H or ^17^O–^2^H DLW using the Coward 1985 two-pools model by the intercept method at the 48 h finals. The blue points are the ^18^O-based results, with 5.1% uncertainty, and the orange points are for ^17^O with 4% uncertainty. The grey points are the individual indirect calorimetry vales for day two (2% uncertainty). **(b)** The difference between the r_CO2_ values calculated by the TLW and IC methods. The r_CO2_ values of the TLW method are the average the r_CO2_ (^18^O) and r′_CO2_ (^17^O) in (**a**). The uncertainty in the difference is around 3.5 ml/h. The values in the plot are the mean ± the SE, for LF, and HF (OR and OP taken together).

The discrepancy between TLW and IC needs an explanation. Figure 6a shows the individual r_CO2_ measured by the 2-1 and 2-2 (Coward 1985) models with the intercept method at 48 h finals, and the individual IC values of day two. It is very clear that r_CO2_ measured based on the ^18^O–^2^H and ^17^O–^2^H pairs are consistent, and their difference is within the uncertainty. For the LF and HF-OP groups, the r_CO2_ (^18^O) is slightly higher than r′_CO2_ (^17^O), but the difference is only about 4 ml/h and not significant. Figure 6b clearly illustrates the deviation of the TLW and IC methods. The r_CO2_ values of the TLW method are calculated by averaging the r_CO2_ (^18^O) and r′_CO2_ (^17^O), and the IC r_CO2_ values are the same as in Fig. 6a. The uncertainty in the difference (TLW minus IC) in Fig. 6b is around 3.5 ml/h (based on 3% relative error for TLW and 2% for IC). The average difference between the TLW and IC values for the LF mice is 7.2 ml/h, while the average distance between TLW and IC for HF groups is 18.1 ml/h, much larger than that of the LF group. The individual IC and TLW data show a similar pattern, which is a firm indication that the difference between TLW and IC is of a systematic nature. As the IC technique is straight forward and less assumption-prone, we suspect the deviation to be caused by isotopic effects not accounted for.

## Discussion

### The triply labelled water method

Because the measurement of δ^17^O has become simple, fast and accurate by the optical spectroscopic system, the classical Doubly Labelled Water (DLW) can easily be extended to Triply Labelled Water (TLW), and to our knowledge we demonstrated that here for the first time. The isotope abundance measurement uncertainties of ^17^O and ^18^O in the blood samples are similar, and the individual turnover rates of ^17^O and ^18^O are also expected to be equal no matter the subject treatment or the turnover time chosen, and our data confirmed this (Fig. 3). The same holds for the dilution space difference between N_17O_ and N_18O_ (Fig. 4). In terms of r_CO2_ calculated based on ^18^O, ^2^H, or on ^17^O, ^2^H, the values also match with each other for the same models (Fig. 5). These findings are consistent with our assumptions: although processes with ^17^O and ^18^O are governed by different fractionation factors, these differences can be accounted for, and do not cause a significant difference in the TLW method calculation. Moreover, using the TLW as extension to DLW, we can double-check the data quality of ^18^O based on the ^17^O data, and lower the measurement uncertainty of the CO_2_ production. In this study, we lower the calculated r_CO2_ uncertainty to 3% when taking the average of r_CO2_ (^18^O, ^2^H, 5%) and r′_CO2_ (^17^O, ^2^H, 4%).

Another use of the third isotope is to help quantify the evaporative water loss fraction, in other words, the TLW method enables us, at least in principle, to derive a direct estimate of the fractionated losses fraction (r_G_). This value of fractional evaporative water losses—r_G_—has been subject of discussion since many decades^4,5,48,49^. As the r_CO2_ (^18^O, ^2^H) should be equal to r′_CO2_ (^17^O, ^2^H), we can derive the individual r_G_ values by equating the two Eqs. (11) and (12). However, the influence of the value of r_G_ is limited: in this paper, we took r_G_ = 0.5, a value that is also widely used in free-living mammals. If we would use r_G_ = 0.25 as other researchers have done^4,48^, the r_CO2_ will increase by less than 2%, which is within the uncertainty band of our TLW average values (3%). Alternatively, one can say that in order to determine r_G_ from the combination (^18^O, ^2^H) and (^17^O, ^2^H) to ± 0.1, one would need an uncertainty in r_CO2_ ≤  ± 1%, out of reach of the present measurement methods, as was already concluded in Ref.^5^.

At the moment, highly enriched ^17^O water is more expensive than pure ^18^O water, this is mainly because of less demand for it. However, ^17^O can now be easily detected by the optical systems such as the one we use. Also, one only needs a factor of 7 less ^17^O label to achieve the same enrichment factor as for ^18^O water, and this reduces the costs. Therefore, adding ^17^O to the classical DLW method is practically easy now, and it is also worthy to use TLW to check the method and improve the precision of the CO_2_ production. At the moment, certified reference waters (such as Ref.^35^) are not yet available for highly enriched ^17^O, so laboratories should make their own references by gravimetrical mixing. However, if demand increases, such reference waters will be made available, by international bodies such as IAEA, or commercial suppliers.

### Calculation protocols

We considered three models for CO_2_ production, one is based on the single-pool model (1-1 and 1-2 in Table 3), another two series of equations are based on the two-pools model (Table 3). In addition, we used the best available values from the literature for the fractionation factors (including ^17^O). The two-pool equations based on Ref.^46^ take the individuals’ specificity more into account, while the equations based on Ref.^45^ use a group average for the dilution space ratio. Therefore, we consider the equations based on Ref.^46^ to be the best form a principal standpoint. Nonetheless, we compared two different group averaging methods for R_dil_ in equations 3-1 and 3-2, one is for the whole group of 18 mice regardless of the feeding methods, the other is separate averages for the three mice subgroups (as used for Fig. 5). Differences in r_CO2_ were less than 2%, so the group difference of R_dil_ is not essential. The results in Figs. 5 and 6 show that the r_CO2_ calculated by Ref.^46^ are indeed closer to the CO_2_ production obtained by indirect calorimetry, but there still is a significant discrepancy, much larger for the 24 h results than for the 48 h ones, and much larger for HF groups than LF groups: The TLW method leads to higher numbers for the r_CO2_.

We found a clear increase from k_2H_-24 h to k_2H_-48 h, but no significant change for k_18O_ and k_17O_. This leads to increased values for r_CO2_ in the first 24 h compared to the second 24 h. One might speculate that this can be caused by the disturbance of the mice during the first day: we injected the labelled water, took the 2 h as well as the 24 h blood samples and put them in and out of the IC box at the first day, but we only took the 48 h samples on the second day. However, the IC results show no significant changes between the first and second day. Due to this low k_2H_-24 h, the TLW r_CO2_ results for the 24 h deviate much more form the IC results than the 48 h results (see Fig. 5). Still, also the 48 h results for rCO2 are high compared to the IC result, which fact one could alternatively formulate as: k_2H_-24 h is much too low, k_2H_-48 is still too low, but by less. If we consider the IC results as straight forward and trustworthy, this would lead to the speculation that the ^2^H label disappears form the body water at a lower rate than the water loss itself, so involving a process with very high fractionation. We discuss this possibility further below, in which discussion the extension of DLW to TLW appears useful.

### Influence of the nutritional conditions

Previous work in our lab focused on different nutrition and body composition effects on r_CO2_ of mice by the DLW technique^13^. They also separated their mice in the same three groups, only their mice were younger. They also found that the r_CO2_ measured by DLW matches IC results much better for low-fat mice than for the high-fat feeding mice, so the high-fat diet is a relevant factor to explain the overestimation of DLW. Intuitively, one might expect that the high-fat fielding mice would consume a higher caloric value of food than the low-fat ones, but this appears to not be the case, rather the opposite (Table 4). However, the individual scatter is large, and does not correlate at all with the individual deviation between IC and TLW values for r_CO2_. Rather, the main difference we consider between our three groups is the turnover rates difference (k_O_–k_2H_), because the TLW-determined body water agrees well with the EchoMRI-determined one, implying that the dilution space for the water is correctly determined. Yet, the r_CO2_ determined by TLW is systematically higher than that with IC, even for the “most agreeing” calculation method (see Figs. 5, 6). This difference is the lowest, regardless of the individual difference, for the LF mice group, close to 8 ml/h, still significantly higher than the largest error of TLW (± 3%) and IC (± 2%). For the HF-OR and HF-OP mice groups, this difference is more than double that amount. The (too) high r_CO2_ values must be caused by too low k_2H_-values, and/or too high k_17O_ and k_18O_ ones. The number of possible explanations is limited, because the body water pool is correctly determined by TLW. The only thinkable way of getting too high ^18^O and ^17^O rates is assuming a strongly fractionating water loss process that preferably takes up ^17^O and ^18^O over ^16^O, and with the same fractionation factor for both. Such a process is next to impossible to imagine, as fractionation factors for ^17^O are typically half those for ^18^O. So, thanks to the extension of the experiments from DLW to TLW, we can now rule that out. For ^2^H on the other hand, we would need an extra water loss or uptake process that heavily discriminates against ^2^H, and such processes are thinkable, and in fact known: electrolysis of water, for example, manifests fractionations of − 600 to − 700‰ (so fractionation factors of 0.4 to 0.3), and also bacterial uptake is known to fractionate considerably (albeit not to the extent of electrolysis). A rough calculation shows that if a 10% extra loss/exchange effect would exist with a ^2^H fractionation factor of 0.4, this would lead to an overestimation of r_CO2_ by 10 ml/h. As the effect is considerably larger for the 24 h than for the 48 h results, the process might in fact be an exchange effect that reaches equilibrium at some point. If that is the case, the TLW-IC difference must gradually disappear. As the discrepancies are much larger for the HF than for the LF mice, body composition (fat content) and/or food digestion must play a profound role in this mechanism.

Identifying the cause of the discrepancy with IC is essential for the reliability of the DLW/TLW methods alike. Thanks to the addition of ^17^O, the mechanism must be some kind of fractionating ^2^H loss. This requires further study, and eventually inclusion of that mechanism into the equations of Table 3.

## Conclusions

This study extends the traditional doubly labelled water technique to triply labelled water to estimate the CO_2_ production for mice held in different nutrition conditions.

The results for both combinations ^2^H–^17^O and ^2^H–^18^O agree well, and hence the calculated r_CO2_ uncertainty is lower and the values are more robust. However, we found that systematic deviations between the DLW (now TLW) method and indirect calorimetry still exist^13^. Thanks to the addition of ^17^O, we can now conclude that a process of extra water removal/uptake with a high degree of discrimination against ^2^H must be the cause of the too high r_CO2_ results. This uptake apparently is dependent on the food intake and/or the body composition. More detailed isotope analysis (such as gastric fluids^50^) can probably reveal this extra water loss/exchange channel.

## Data Availability

The study is reported in accordance with ARRIVE guidelines (https://arriveguidelines.org). All data described in the manuscript are in the database system of the Centre for Isotope Research, and are available upon request.

## Code availability

The custom-made R data processing program described in the manuscript is available upon request.
